# Effect of chronic administration of arachidonic acid on the performance of learning and memory in aged rats

**DOI:** 10.29219/fnr.v63.1441

**Published:** 2019-03-25

**Authors:** Takayuki Inoue, Michio Hashimoto, Masanori Katakura, Shahdat Hossain, Kentaro Matsuzaki, Osamu Shido

**Affiliations:** 1 Department of Environmental Physiology, Shimane University Faculty of Medicine, Izumo, Shimane, Japan; 2 Department of Biochemistry and Molecular Biology, Jahangirnagar University, Savar, Dhaka, Bangladesh

**Keywords:** arachidonic acid, spatial learning, senescent, reference and working memory, radial maze

## Abstract

**Background:**

Arachidonic acid (AA, C20:4, ω-6) is a ω-6 polyunsaturated fatty acid (PUFA) and plays diverse roles in cell signaling. Numerous reports on the effects of ω-3 PUFAs, such as docosahexaenoic acid (DHA, C22:6, ω-3) and eicosapentaenoic acid (EPA, C20:5, ω-3) on learning and memory impairments of rats are available, however, the role of AA on brain cognition is largely unknown.

**Objective:**

In this study, our aim was to investigate the effect of oral administration of AA on spatial memory-related learning ability in aged (100 weeks) male rats.

**Design:**

One group was per orally administered 240 mg/kg per day AA oil and the other group was administered the similar volume of control oil. Five weeks after the start of the administration, rats were tested with the partially baited eight-arm radial maze to evaluate two types of spatial memory-related learning ability displayed by reference memory errors (RMEs) and working memory errors (WMEs). Also, the time required to complete the task was recorded. The levels of lipid peroxide (LPO) and reactive oxygen species (ROS) were measured, as an indicator oxidative stress in the plasma and brain corticohippocampal brain tissues.

**Results:**

The scores of RMEs and WMEs, which are analogous to long-term and short-term memory, respectively, were not affected, however, the trial time was shorter in the AA-administered rats than that of the controls. AA also significantly increased the degree of oxidative stress both in the plasma and corticohippocampal brain tissues.

**Conclusions:**

Our results suggest that though AA deposition in the corticohippocampal tissues of senescent rats caused a faster performance activity, which is reminiscent to hyperactive behavior of animals, the spatial learning ability-related memory of the rats, however, was not improved.

## Popular scientific summary

- We found that oral administration of arachidonic acid (AA, C20:4, ω-6) to aged rats did not affect the spatial learning-related memory, as evaluated in the eight-arm radial maze.
- Compared to control animals, the AA-administered rats, however, completed the maze task at a significantly faster pace, i.e.. the trial time was shorter.
- Increases in the AA levels in the corticohippocampal brain regions or plasma were accompanied with increased oxidative stress in these tissues.
- We recommend that the purpose of the memory-enhancing effect of AA in the elderly needs re-evaluation, as the faster performance activity in many instances is considered reminiscent of hyperactive behavior of animals.

Arachidonic acid (AA, C20:4, ω-6) − a ubiquitous polyunsaturated fatty acid (PUFA) present in all mammalian cells – can be produced *in vivo* from its precursor ω-6 linoleic acid (LLA, C18:2, ω-6) and is also normally consumed in small amounts in the regular diet (1). As opposed to docosahexaenoic acid (DHA, C22:6, ω-3), for which the brain is the most active retention site (2), AA is actively retained in the skeletal muscle, where it accounts for roughly 10–20% of the phospholipid fatty acid content (3). AA is also referred to as a conditionally essential fatty acids for animals (4–6), including humans, that experience persistent deficiencies of LLA (C18:2, ω-6), or during prematurity, growth, or if there is a limited capacity to convert LLA to AA (4). However, consumption of vegetable-based oils with large amounts of LLA and an adequate capacity to convert LLA to AA can eliminate the need for exogenous AA and thereby eliminate its role as an essential fatty acid. In addition to its involvement in bilayer membranes as a structural fatty acid, AA may act as a direct substrate for the conversion of lipid mediators, namely eicosanoids (7, 8), through cyclooxygenase, lipoxygenase (LOX), and P-450 epoxygenase pathways (9–11). However, AA-derived eicosanoid mediators have established roles in inflammation in tissues, and AA metabolism is a well-known target for commonly used anti-inflammatory therapies (12, 13). Excessive consumption of AA can result in increased platelet aggregation and vasoconstriction by thromboxane A2, which can lead to thrombogenesis and myocardial infarction (13–15). In contrast, however, several clinical studies have also reported that the use of AA supplements showed no significant adverse effects on kidney or liver function, serum lipids, immunity, or platelet aggregation (16). Rather, a higher concentration of AA in muscle tissue was associated with improved insulin sensitivity (17).

Although many neuroscientists have investigated the effect of DHA on cognitive aptitude in older individuals and patients with Alzheimer’s disease (AD), research on the effect of AA on learning and memory is limited. Although some noteworthy *studies have* been published, their results have not been conclusive. For instance, whether AA itself or AA-derived lipid mediators are responsible for changes in the brain underlying changes in cognition remains to be determined. Intrahippocampal injection of prostaglandin E2, an AA cascade product, has been shown to impair working memory (18). On the contrary, long-term dietary administration of AA to senescent rats has been reported to heighten hippocampal long-term potentiation (LTP) (19), the neurochemical foundation of learning and memory. AA has also been reported to limit learning and memory impairments in rats by ameliorating cholinergic dysfunctions (20). All these reports demonstrate that an appreciation for the role of brain AA in cognition continues to grow, but also remains to be clarified.

Compared with young rats, elderly animals usually show a significant decrease in cognitive functions (21). This decline has been attributed to age-dependent reduction in membrane PUFAs, especially DHA and AA, which are abundant in hippocampal neurons (22, 23). In a previous study, we reported that chronic administration of DHA was effective in improving spatial learning and memory (24, 25). Furthermore, we reported that chronic oral administration of highly concentrated DHA, together with eicosapentaenoic acid (EPA, 20:5n-3), promoted an antioxidative effect in brain corticohippocampal tissue in a rat model of AD, concurrently with improvements in learning and memory (2, 26, 27). We also reported that chronic oral administration of AA significantly decreased the anti-inflammatory compound ω-3 DHA (and also ω-3 EPA-derived eicosanoids) in the rat kidney (28) and that oxidative stress significantly increased in plasma (29). As there are a limited number of reports describing the effect of chronic exogenous administration of AA on learning and memory, and in an effort to extend our previous findings, we evaluated the effect of chronic oral administration of AA on cognitive function in old rats in this study.

## Materials and methods

### Animals

Five-week-old Wistar (Jcl:Wistar) rats (Generation 0; G0) obtained from Clea Japan (Osaka, Japan) were housed and maintained in an air-conditioned room under a 12-h/12-h light/dark cycle and provided fish oil-deficient food (F1; Funabashi Farm, Funabashi, Japan) and water, *ad libitum*. Breeding commenced when the animals were 3 months of age. Pups (G1 and G2) were maintained under the same conditions as the G0 rats. G2 aged rats were used for this study, which was conducted under the procedures outlined in the *Guidelines for Animal Experimentation* of Shimane University, compiled from the *Guidelines for Animal Experimentation* of the Japanese Association for Laboratory Animal Science.

### AA administration

The aged G2 male rats (100 weeks old) were randomly divided into AA and control groups. Rats in the AA group were administered AA oil intragastrically (Triglyceride (TG) form of AA-rich oil; 240 mg/kg body weight [BW]/day) (Table 1). The AA oil was gently emulsified in an ultrasonic homogenizer (Taitec VP-5; Taitec, Tokyo, Japan) immediately before administration. The control group was administered a similar volume of control oil (beef fat, soybean oil, and rapeseed oil, in a 2:1:1 ratio) lacking AA. Administration of both oils was maintained until all experiments were completed. Table 1 shows the composition of the fatty acids in each oil.

**Table 1:** Composition of fatty acid administered to the rats

	Control oil	Arachidonic acid oil
Palmitic acid, C16:0, PLA	13.80 ± 0.01	6.95 ± 0.00
Stearic acid, C18:0, STA	13.80 ± 0.01	5.91 ± 0.00
Oleic acid, C18:1,ω-9, OLA	42.50 ± 0.03	5.31 ± 0.00
Linoleic acid, C18:2ω-6, LLA	20.00 ± 0.02	9.38 ± 0.01
Arachidonic acid, C20:4,ω-6, AA	ND	45.10 ± 0.04
Eicosapentaenoic acid, C20:5,ω-3, EPA	0.13 ± 0.01	0.52 ± 0.00
Docosapentaenoic acid, C22:5,ω-3, DPA	Not detected (ND)	ND
Docosahexaenoic acid, C22:6,ω-3, DHA	ND	ND

Results are mean ± SEM, for triplicate determinations.

### Eight-arm radial maze task

Behavioral testing was performed in an eight-arm radial maze, as previously described (26, 27). Briefly, the maze consists of an octagonal central platform surrounded by eight equally spaced radial arms. A food well is located at the end of each arm. The maze was placed in a closed room, with visual cues for orientation. The experimenter maintained a constant position beside the maze and observed the behavior of the rats. Six weeks after initiating AA administration, rats were placed on a food deprivation regimen that reduced BW to 70–75% of the free-feeding weight and were then handled 5 min daily for 5 consecutive days. Subsequently, for 5 additional days, rats were familiarized with the testing apparatus where 45 mg reward pellets (made with the F1 food) were scattered throughout the maze.

Each rat was tested during two trials per day, 6 days per week, for a total of 3 weeks. A trial consisted of baiting four of the eight arms (consistently using the same four arms for a given animal) with reward pellets and subsequently placing the rat on the central platform facing a randomly selected arm. The test assessed two parameters of learning and memory: 1) reference memory errors (RMEs), evidenced by entry into unbaited arms; and 2) working memory errors (WMEs), evidenced by repeated entry into arms that had already been visited within a trial.

### Blood and brain preparation

Following the completion of behavioral testing, rats were deeply anaesthetized by an intraperitoneal injection of pentobarbital (65 mg/kg BW) and blood was drawn for the biochemical assays. The cortex and hippocampus were dissected from each rat for subsequent analysis. Some samples were rapidly dissected and immediately frozen in liquid nitrogen for further analysis.

### Lipid analysis

Samples were homogenized with a Polytron homogenizer (PCU-2-110; Kinematica GmbH, Steinhofhalde, Switzerland) in phosphate-buffered saline (1 mL/100 mg tissue) that contained 0.005% (w/v) 2,6-di-t-butyl-4-methylphenol (Wako Chemicals, Osaka, Japan). Protein concentrations were estimated using the method described in Lowry et al. (30). Fatty acids in the plasma and muscles were prepared and analyzed by a modification of the one-step reaction analysis suggested by Lepage and Roy (31) using gas chromatography (GC) (2, 27). For each sample, the mixture of plasma or brain tissue homogenate was augmented with 2 mL of methanol containing 10 μg of tricosanoic acid as an internal standard and 200 μL of acetyl chloride. This mixture was then incubated at 100°C for 60 min, followed by the addition of 200 μL of octane and 5 mL of 10% sodium chloride containing 0.5 N sodium hydroxide. The mixture was shaken for 10 min at room temperature and centrifuged at 2,800 × g for 15 min. The octane phase, which contained the fatty acid methyl esters, was subjected directly to GC in the Agilent 6850A gas chromatograph (Agilent Technologies, Santa Clara, CA, USA).

### Antioxidative–oxidative status

Lipid peroxidase (LPO) concentration was assessed by the thiobarbituric acid reactive substance assay designed by Ohkawa et al. (32), as previously described (27), and its levels were expressed in nanomoles of malondialdehyde per milligram of protein. Malondialdehyde levels were calculated relative to a standard preparation of 1,1,3,3-tetraethoxypropane. The level of reactive oxygen species (ROS) was determined as previously described (2, 26, 27). Briefly, 50 μL of freshly prepared tissue homogenate was diluted in 4.85 mL of 100 mmol/L potassium phosphate buffer (pH 7.4) and incubated for 15 min at 37°C with dichlorofluorescein diacetate (Sigma-Aldrich, St. Louis, MO, USA) in methanol, at a final concentration of 5 μmol/L. The dye-loaded samples were centrifuged at 12,500 × g for 10 min, at 4°C. The pellet was vortexed at 4°C in 5 mL of 100 mmol/L phosphate buffer (pH 7.4) and incubated again for 60 min at 37°C. Fluorescence was measured with a Hitachi 850 spectrofluorometer at wavelengths of 488 nm for excitation and 525 nm for emission. The cuvette holder was maintained at 37°C. ROS levels were quantified from the dichlorofluorescein standard curve in methanol.

### Statistical analysis

**Fig. 1:**
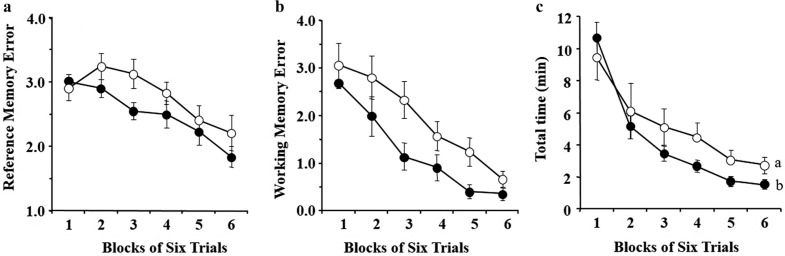
Eight-arm radial maze task. (a) Effect of chronic administration of AA on the number of Reference Memory Errors (RMEs) in aged rats. (b) Effect of chronic administration of AA on the number of working memory errors (WMEs) in aged rats. (c) Effect of chronic administration of AA on the trial time in aged rats. Data are the mean ± SEM. Groups without a common letter are significantly different at *p* < 0.05. Each value represents the number of RMEs averaged over six trials. The data were analyzed by randomized two-factor (block and group) ANOVA followed by Fisher’s protected least significant difference test for *post hoc* comparisons. AA, arachidonic acid.

Results are expressed as mean ± standard error of the mean (SEM). Behavioral data (Fig. 1) were analyzed by a randomized two-factor (group and block) block factorial analysis of variance (ANOVA), while all other parameters (Table 2) were analyzed for intergroup differences by one-way ANOVA. ANOVA was followed by the Fisher’s protected least significant differences test, for *post hoc* comparisons. Correlations were determined using simple regression analysis (Table 3). GB-STAT™ 6.5.4 (Dynamic Microsystems, Inc., Silver Spring, MD, USA) and StatView 4.01 (MindVision Software, Abacus Concepts, Inc., Berkeley, CA, USA) were used for statistical analyses. Statistical significance was set at *p* < 0.05.

**Table 2:** Fatty acid profile and oxidative status in plasma

	Control (*n* = 7)	AA (*n* = 8)
PLA C16:0	24.34 ± 0.46	22.73 ± 0.35**
STA, C18:0	14.21 ± 0.23	14.55 ± 0.28
OLA,C18:1,ω-9	10.30 ± 0.62	8.58 ± 0.48**
LLA, C18:2,ω-6	18.02 ± 1.05	14.02 ± 0.67**
AA, C20:4,ω-6	28.64 ± 1.62	36.67 ± 1.30**
EPA, C20:5,ω-3	0.71 ± 0.10	0.35 ± 0.07**
DPA, C22:5,ω-3	0.53 ± 0.22	0.44 ± 0.05
DHA, C22:6,ω-3	1.78 ± 0.23	1.27 ± 0.19*
ω-6/ω-3	15.51 ± 2.45	24.55 ± 2.68**
DHA/AA	0.06 ± 0.01	0.04 ± 0.01**
EPA/AA	0.03 ± 0.00	0.01 ± 0.00**
Unsaturation index	179.09 ± 3.62	195.84 ± 2.82**

Results are mean ± SEM. Values in the same row with different symbols are significantly different at

** *p* < 0.05,

* 0.05 < *p* < 0.1 (unpaired Student’s *t*-test). Unsaturation index was calculated as [Σ(mole% of each unsaturated fatty acid × number of double bonds/fatty acid)]/100.

**Table 3:** Fatty acid profile and oxidative status in the brain cortex and hippocampus

	Cerebral cortex		Hippocampus	
	Control (*n* = 7)	AA (*n* = 8)	Control (*n* = 7)	AA (*n* = 8)
PLA, C16:0	26.59 ± 0.47	27.03 ± 0.30	25.39 ±0.34	25.59 ± 0.18
STA, C18:0	27.70 ± 0.29	27.98 ± 0.12	26.52 ± 0.14	26.64 ± 0.13
OLA, C18:1,ω-9	18.96 ± 0.71	18.10 ± 0.51	20.90 ± 0.46	20.41 ± 0.30
LLA, C18:2,ω-6	0.80 ± 0.05	0.58 ± 0.05**	0.63 ± 0.04	0.49 ± 0.02**
AA, C20:4,ω-6	11.59 ± 0.37	12.72 ± 0.35**	12.77 ± 0.20	13.37 ± 0.14**
EPA, C20:5,ω-3	0.07 ± 0.01	0.06 ± 0.00*	0.09 ± 0.00	0.08 ± 0.01
DPA, C22:5,ω-3	0.11 ± 0.01	0.09 ± 0.01**	0.11 ± 0.01	0.11 ± 0.01
DHA, C22:6,ω-3	13.04 ± 0.45	12.47 ± 0.20	11.84 ± 0.20	11.54 ± 0.20
ω-6/ω-3	0.94 ± 0.05	1.06 ± 0.04*	1.11 ± 0.02	1.18 ± 0.02**
DHA/AA	1.13 ± 0.06	0.99 ± 0.04*	0.93 ± 0.02	0.86 ± 0.01**
EPA/AA	0.01 ± 0.00	0.00 ± 0.00**	0.01 ± 0.00	0.01 ± 0.00*
Unsaturation index	146.83 ± 2.06	146.36 ± 0.67	146.45 ± 1.18	146.19 ± 1.08

Results are mean ± SEM. Values in the same row (either of the cortex or hippocampus) that differ with common symbols are significantly different at

** *p* < 0.05,

* 0.05 < *p* < 0.1 (unpaired Student’s *t*-test). Unsaturation index was calculated as [Σ (mole% of each unsaturated fatty acid × number of double bonds/fatty acid)]/100.

## Results

### Body weight

Body weight was not affected by oral administration of AA.

### Eight-arm radial maze task

The effect of chronic administration of AA on reference and working memory-related tasks is shown in Fig. 1a and 1b. The effect of chronic administration of AA on trial duration is shown in Fig. 1c. Randomized two-factor (block and group) ANOVA, to analyze the effect of AA, revealed significant main effects of both the blocks of trials (*p* < 0.0001) and the groups (RME; *p* < 0.01, WME; *p* < 0.0001) on the number of RMEs (Fig. 1a) and WMEs (Fig. 1b), but there was no significant block × group interaction. Interestingly, a significant main effect of blocks of trials (*p* < 0.0001) was observed with no significant main effect of groups of trials (*p* = 0.0728) on the length of trial duration, but with a significant block × group interaction (*p* = 0.0011) (Fig. 1c).

### Fatty acid profiles and oxidative status of the plasma

**Fig. 2:**
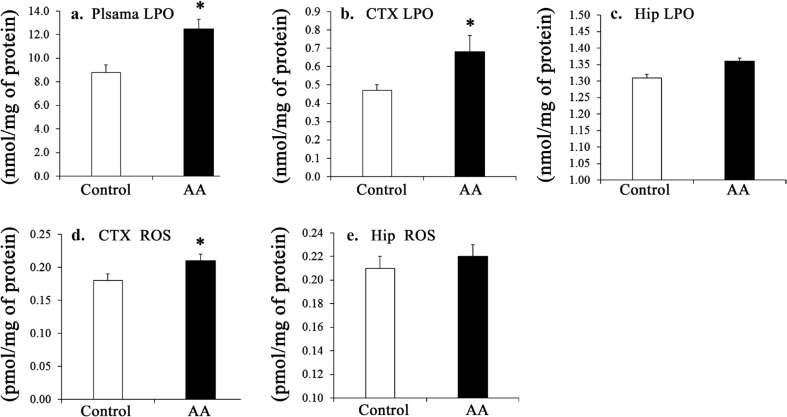
Effect of chronic administration of AA on LPO of the plasma (a), cortex (b), and hippocampus (c), and those on the cortex (d) and hippocampus (e) ROS. Results are mean ± SEM, *n* = 7~8. Significant difference at *p < 0.05. AA, arachidonic acid. LPO, Lipid peroxide; ROS, Reactive oxygen species; CTX, Cortex; Hip, Hippocampus.

In the plasma, the AA level, the ω-6/ω-3 ratio, and the unsaturation index were significantly higher in the AA group than in the control group (*p* < 0.05), while levels of palmitic acid, oleic acid, linoleic acid (LLA), and EPA, as well as the DHA:AA and EPA:AA ratios, were significantly lower in the AA group (*p* < 0.05) (Table 2). The plasma LPO level was significantly higher in the AA group (*p* < 0.05) (Fig. 2a).

### Fatty acid profiles and oxidative status in the cerebral cortex and hippocampus

In the cerebral cortex, the AA level was significantly higher in the AA group than in the control group (*p* < 0.05). LLA, docosapentaenoic acid (DPA) levels, and the EPA:AA ratio were significantly lower in the AA group (*p* < 0.05) (Table 3), while the ROS level was significantly higher in the AA group (*p* < 0.05) (Fig. 2b and 2d). In the hippocampus, the AA level and the n-6:n-3 ratio were significantly higher in the AA group (*p* < 0.05) and the LA levels and the DHA/AA ratio were significantly lower (*p* < 0.05) (Table 3).

Very significant positive correlations were observed between the plasma LLA and EPA levels and between the LLA and the DHA:AA ratio in the hippocampus (Table 4). Significant negative correlations were also observed between the plasma LLA and AA levels and also the LLA and AA levels in the hippocampus (Table 4). Similarly, a very significant negative correlation was also observed between the plasma EPA level and the ω-6:ω-3 ratio in the hippocampus (Table 4). Correlations were not observed between the plasma LLA, AA, or EPA levels, and the LLA, AA, DPA levels or the EPA:AA ratio in the cerebral cortex (data not shown). Significant negative correlations were also observed between plasma LPO levels and the EPA:AA ratio in the cerebral cortex (*r* = –0.560, *p* = 0.030).

**Table 4:** Correlation coefficients between fatty acid in plasma and fatty acid in hippocampus

Plasma fatty acids	Hippocampus			
	Linoleic acid (LLA) (mol%)	Arachidonic acid (AA) (mol%)	w-6:w-3	DHA:AA (docosahexaenoic acid:arachidonic acid)
LLA (mol%)	0.734 (0.002)	−0.678 (0.006)	Not significant (N.S.)	0.555 (0.032)
AA (mol%)	−0.587 (0.021)	N.S.	N.S.	N.S.
Eicosapentaenoic acid (EPA) (mol%)	0.656 (0.008)	N.S.	−0.669 (0.006)	0.721 (0.002)

Results are evaluated with simple regression analysis. *P*-values are expressed inside the parentheses.

## Discussion

We previously reported that dietary DHA (C22:6, ω–3) enhances learning and memory in young (24) and old (25) rats, as well as rat models of AD (2). Because AA (C20:4, ω-6) is the second most abundant PUFA in the brain, in the current study, we assessed the effect of chronic oral administration of AA on spatial learning and memory in senescent rats. The results of the current study clearly suggest that chronic oral administration of AA does not significantly alter spatial working or reference memory, as evaluated in the eight-arm radial maze task. However, oral administration of AA did result in a significant decrease in ‘trial time’ − the time during which each rat was tested to collect the food rewards located at the ends of the radial maze arms. In other words, compared to control animals, AA-treated rats completed the maze task at a significantly faster pace (shorter time). Specifically, the AA-treated rats were found to be more active during the radial maze task in our experimental paradigm. Despite this improved performance with respect to timescale in the radial maze, the RME and WME scores of the AA rats were not significantly different from those of controls throughout the experimental sessions. However, the behavioral performance was accompanied by an increase (*p* < 0.05) in the levels of AA in the plasma, cerebral cortex, and hippocampus. In contrast, levels of hippocampal (phospholipid) AA were unchanged in a similar study, where AA administration significantly increased spatial cognition and improved memory performance, measured in the Morris water maze (33). Escape latencies, the time required to reach the target platform in the Morris water maze of AA-fed rats, were also shorter, just as the AA-fed rats consistently took less time to complete the eight-armed maze task in our study. Despite the faster completion of the radial maze task, why AA administration did not affect the WME or RME scores (short-term and long-term memory) remains to be determined. This also raises the question of whether AA enrichment in the brains of senescent rats functions directly to improve memory performance or if perhaps instead it is required to facilitate signal transduction necessary for other brain functions, including neuro-immunomodulation. The enhancement of AA in corticohippocampal tissues made the AA rats, by some means, more active, which may relate to muscular activities associated with mental processes. One potential mechanism by which AA might contribute to shorter trial times is via an increase in motor neuron excitability, occurring through an increase in eicosanoids that then enhances the release of neurotransmitters at the neuromuscular junction (34).

Our data were, however, consistent with a study in which AA concentration in hippocampal cell membranes was significantly higher in AA-treated aged rats, compared with age-matched rats fed a control diet (22). Recently, Li et al. (20) also reported that chronic oral administration of AA caused a reduction in isoflurane-induced impairments in the Morris water maze task (20). Matsumoto et al. (18) reported that an AA cascade product, specifically, prostaglandin E, causes an impairment of working memory, thus supporting the proposition that AA is likely not directly involved in the formation of memory. After chronic administration of DHA for 12 weeks, Gamoh et al. (24) reported that the AA was negatively correlated with memory. Thus, we speculate that the mechanism underlying AA-induced modulation of memory is not straightforward; rather, a complex mechanism may be involved. Alternatively, discrepancies might be related to differences in AA dosing in our study (~55 mg AA/rat) relative to other studies (40 mg AA/rat), differences in the rat strains tested, or in the tasks utilized to assess learning and memory (radial maze vs. a water maze). For example, a radial maze involves a search – that is, navigation within highly structured, predetermined, and constrained routes (arms) – while a water maze requires the animal to navigate via an unconstrained route where water acts as a negative stimulus. Sensory cues (water vs. air) are different; hence, sensory inputs would also be different. Thus, the learning and memory tasks required for the water maze and the radial maze, even in the same animal, might be consistently different and are not necessarily correlated. Nonetheless, it remains to be determined why increased levels of AA in the plasma, cortex, and hippocampus did not improve memory in the AA-administered rats in our present study. Moreover, it remains to be clarified how AA affects information processing in motor systems of the brain and how coordination with the skeletal system is achieved in order to achieve faster trial times.

AA has also been inferred to be detrimental for the induction of neuro-inflammation leading to neurodegenerative diseases, such as AD patients, rat and mouse model (35–37). Tumor necrosis factor alpha (TNFα)-driven inflammation may have a deleterious effect on neurons (38). Challenging primary cultures of rodent and human microglial cells with Aβ induces the release of high levels of TNFα (39, 40). More importantly, AD postmortem frontal cortex samples showed increased levels of TNFα and AA cascade products (41). Inhibition of soluble TNFα signaling in a mouse model of Alzheimer’s disease prevented Aβ-associated neuropathology. Therefore, targeting Tumor necrosis factor alpha (TNFα) has been a therapeutic strategy for AD (42). Though we did not measure the levels of TNFα in the rat brains of the present investigation, we could not find any effects of oral administration of AA on the levels of TNFα and other interleukin (IL) cytokines, including IL-1b, IL-6, IL-4, IL-10, and IL-in the plasma.

Furthermore, the increased levels of brain LPO and ROS (Fig. 2), indicators of oxidative stress, cause neurodegeneration, leading to a decay of memory during aging (34). Thus, it is not unlikely that the AA-induced oxidative potential may have offset the effects of AA on memory enhancement in the AA-fed rats in our study. In contrast, levels of LPO and ROS in the muscle (29) and kidney (28) were not changed. This suggests a differential effect of AA on oxidative stress in the brain versus other tissues, including muscle and kidneys. Memory in normal aged rats (25) and in AD model rats (2) was negatively associated with AA. Yet, AA, in the triacylglycerol form, improved cognitive function in the elderly only at low serum levels of AA (43); thus, we consider that AA appeared as a conditional essential PUFA in elderly subjects in this investigation. Other studies have investigated possible mechanisms of action for AA. Membrane AA, after its release and conversion by phospholipase A2 (PLA2) and LOX, respectively, into eicosanoids (e.g. hydroxyeicosatetraenoic acids (HETEs) and hydroperoxyeicosatetraenoic acids (HPETEs), has been proposed to act as a retrograde messenger to sustain LTP in the hippocampus (44). In contrast, reduced levels of membrane AA, as manipulated by DHA supplementation, were not likely to be a causal factor in the decline of LTP (45). In aged rats, supplemental vitamin E reversed the LTP deficits, associated with an increased membrane lipid peroxidation, at the expense of membrane AA (46), indicating that maintenance of LTP is a multifactorial phenomenon and that membrane AA is not the sole agent responsible for the establishment and maintenance of LTP.

Finally, levels of LPO significantly increased in the plasma, cortex, and hippocampus of the AA-administered senescent rats. Cognitive decline during aging is linked to the generation of ROS, oxidative stress, and decreased synaptic plasticity. Mechanisms to remove oxidized cells and tissues decrease with aging. Therefore, our results indicate that all these factors must be considered for the supplementation of AA in aging subjects.

## Conclusion

Our experiments suggest that although AA deposition in the cerebral cortex or hippocampus of aged rats induces faster performance activity, spatial learning and memory do not improve.
